# OLDER ADULTS’ PERSPECTIVES ON USING TECHNOLOGY TO ASSESS SOCIAL ISOLATION AT THE ONSET OF COVID-19

**DOI:** 10.1093/geroni/igad104.3740

**Published:** 2023-12-21

**Authors:** Jarshini Nanthakumar, Karishma Patel, Shahrose Aratia, Priyanka Thakkar, Rosalie Wang, Shehroz Khan

**Affiliations:** University of Toronto, Toronto, Ontario, Canada; University of Toronto, Toronto, Ontario, Canada; University of Toronto, Toronto, Ontario, Canada; University of Toronto, Toronto, Ontario, Canada; University of Toronto, Toronto, Ontario, Canada; Toronto Rehabilitation Institute, Toronto, Ontario, Canada

## Abstract

Social isolation (SI) among older adults (OA) is a global health issue that was exacerbated by the pandemic. This study examines OA’s perspectives on technology use (i.e., home-based ambient sensor or wearable technology) to assess SI, with consideration of understanding these perspectives in relation to the pandemic. Two questions were examined: (i) How familiar are OA with technology that can be used to assess SI? and (ii) Are OA agreeable to using technology to assess SI? Online surveys (n=4379) and semi-structured interviews were completed by community-dwelling OA at the onset of COVID-19. Quantitative data were analyzed using descriptive statistics and qualitative data were analyzed using content analysis. From survey responses, 16.79% indicated they would be agreeable to using technology to assess SI, whereas 40.37% and 42.83% indicated no and maybe/not sure, respectively. Although most participants were familiar with some technologies that could be used as part of an SI assessment tool, interviews suggested they were unfamiliar with this application of technologies. Preliminary analysis of the interviews (n=31) revealed three broad categories addressing the research questions: (1) perceived barriers to sensor-based technology adoption, (2) user experience and acceptance, and (3) changing social norms. Our study is one of the first to examine the views of OA regarding familiarity and openness to technology use for SI assessment that emerged with COVID-19. Technology developers and healthcare providers need to address ethical concerns around sensor-based technology use and increase opportunities to improve OA’s digital literacy and accessibility to support technology acceptance for SI assessment.

